# Nootkatone inhibits the progression of glioblastoma by activating the ATF4-CHOP-CHAC1 pathway

**DOI:** 10.1186/s10020-025-01064-1

**Published:** 2025-01-16

**Authors:** Qian Wang, Xiumin Xue, Zhichao Chen, Wei Zhang, Yiming Qian, Danni Chen, Lin Lin, Yinfeng Yuan, Weiqiao Zhao, Zhihui Huang, Yongjie Wang

**Affiliations:** 1 School of Pharmacy, Hangzhou Normal University, Hangzhou, 311121 China; 2 Key Laboratory of Elemene Class Anti-Cancer Chinese Medicines, Engineering Laboratory of Development and Application of Traditional Chinese Medicines, Collaborative Innovation Center of Traditional Chinese Medicines of Zhejiang Province, Hangzhou Normal University, Hangzhou, 311121 China

**Keywords:** Nootkatone, Glioblastoma multiforme, Proliferation, ROS, ATF4-CHOP-CHAC1

## Abstract

Glioblastoma multiforme (GBM) represents a primary brain tumor that is widely prevalent, and clinical drugs available for its treatment exhibit varying degrees of resistance. Nootkatone (NKT) is a functional sesquiterpene sourced from traditional Chinese medicine --Alpinia Oxyphylla Miq and has been reported to have a diverse range of pharmacological properties. However, it remains unknown whether there are effects of NKT on GBM. In this study, we found that NKT inhibited the growth of GBM cells in a dose-dependent manner in vitro. Subsequently, we observed that NKT suppressed the migration and arrested cell cycle at G2/M phase of GBM cells. Furthermore, NKT induced the death of GBM cells accompanied by an increase in reactive oxygen species (ROS) production. Mechanistically, we found that NKT inhibited the progression of GBM cells through activating the ATF4-CHOP-CHAC1 pathway in GBM cells. Furthermore, NKT-induced inhibition of migration and proliferation in GBM cells was partially restored by ATF4 or CHAC1 knockdown. Finally, we found that NKT inhibited the growth of tumor in GBM orthotopic mice model through activation of ATF4-CHOP-CHAC1 axis. Taken together, our findings show that NKT suppresses the growth and migration of GBM cells by activating the ATF4-CHOP-CHAC1 pathway, which in turn prevents the tumorigenesis of GBMs and provides a novel perspective for the development of drugs against GBM.

**Graphical Abstract:**

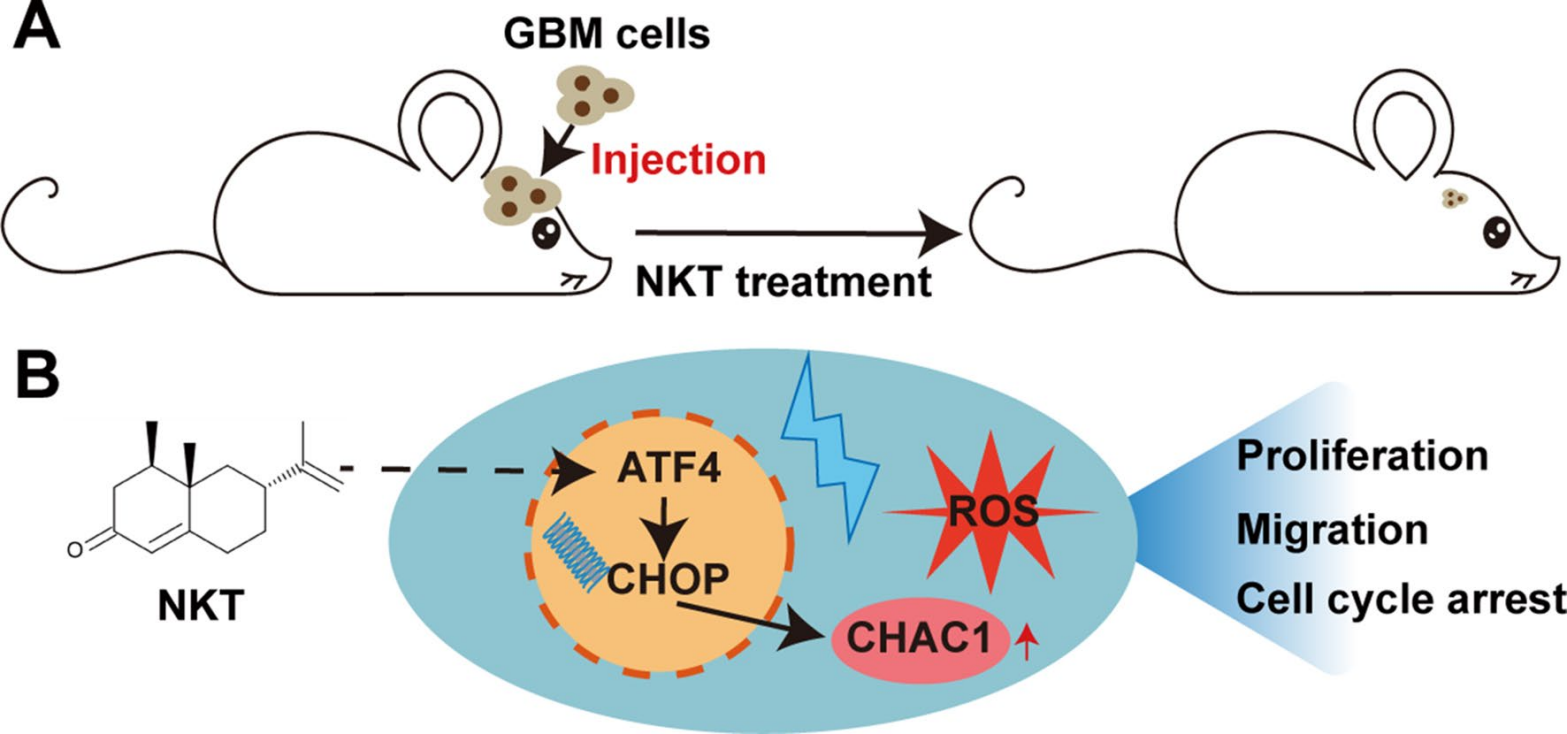

**Supplementary Information:**

The online version contains supplementary material available at 10.1186/s10020-025-01064-1.

## Introduction

Glioblastoma multiforme (GBM) ranks among the most prevalent primary brain tumor (Wirsching et al. 2016), and its five-year case-fatality rate is surpassed only by pancreatic and lung cancers among systemic tumors (Bai et al. 2022). Due to the specificity of tumor anatomic location and the invasiveness of growth, combined with surgical recurrence, GBM patients have a median survival rate of only 12–15 months and a 5-year survival rate of less than 5% (Perry et al. 2017; Li et al. 2021). The complex mechanism of drug resistance in current GBM therapy results in a decline in the quality of life of GBM patients, though traditional remedies can prolong the lifespan to some extent (Li et al. 2020). Therefore, there is a pressing need to develop novel, safe and effective therapeutic agents that can improve both the survival rate and quality of life of GBM patients.

In recent years, natural drugs exhibiting potential anti-neoplastic properties have garnered much attention in the realm of cancer prevention and treatment (Zhu et al. 2023; Zhang et al. 2021; Sui et al. 2020a, b). Numerous natural bioactive substances obtained from microorganisms, fungi, plants and animals have been utilized in clinical or clinical trials (Shi et al. 2022; Sun et al. 2022; Zhai et al. 2020). The application of biological extracts as avant-garde and efficacious interventions for cancer holds great potential (Zou et al. 2024; Jiang et al. 2024). Nootkatone (NKT), a functional sesquiterpene retrieved from *Alpinia Oxyphylla Miq*, a constituent of traditional Chinese medicine (Wang et al. 2022a, b), has been reported to elicit various pharmacological effects, such as anti-inflammatory activity, mitigate Alzheimer’s disease and neuroprotective effects (MacLeod and Buigues 1964; Yu et al. 2022). Recently, NKT has shown anti-tumor activity in several studies (Hung et al. 2019) and has been demonstrated to augment the expression of NAG-1 expression and attenuate the expression of Cyclin D1, and inhibit the proliferation of colorectal cancer cells (Yoo et al. 2020). These findings suggest that NKT may have a role in anti-tumor activity, however, its contributions to the development of anti-GBM remain uncovered.

To examine this hypothesis, we conducted a series of experiments to evaluate the anti-GBM effects of NKT both in vitro and in vivo. We discovered that NKT activated the ATF4-CHOP-CHAC1 pathway to induce the death of GBM cells and inhibit their growth and migration. Our research provides a promising candidate for creating a cancer treatment for GBM.

## Materials and methods

### Cell culture and animals

Prof. Maojin Yao (Sun Yat-Sen University, Guangdong, China) kindly donated the GBM cell lines C6 and U251, which were grown aseptically at 37 °C in a humid incubator with 5% CO_2_, and the murine GBM cell line GL261 was purchased from Tongpai (Shanghai, China) biotechnology Co., Ltd. Gempharmatech Co., Ltd. provided male C57BL/6 mice that were 6 to 8 weeks old and weighed an average of 22 g. They had a 12 h light/dark cycle, stable room temperature and humidity, normal air (21% O_2_), and regular food in room air. The Animal Care and Use Committee at Hangzhou Normal University granted consent to all animal research, and the approval number is HSD20211003.

### Drugs

Nootkatone was purchased from MedChemExpress (#HY-N2195). The solid powder was dissolved with DMSO to a concentration of 458.02 mM and stored at -80 °C.

### GEPIA analysis

Gene expression profiling interactive analysis (GEPIA) (http://gepia.cancer-pku.cn/index.html) database was applied to re-identify the expression of CHAC1 in normal and GBM. The database was a web server for analyzing the RNA sequencing expression data of 207 normal and 163 tumor samples from the TCGA and the GTEx projects (Tang et al. 2017).

### Molecular docking simulation

The 3D structure of ATF4 (PDB ID: 6IRR) (Wang et al. 2021) was from the Protein Data Bank (PDB) (https://www.rcsb.org). The structure of NKT was obtained in a two-dimensional structure format from PubChem (https://pubchem.ncbi.nlm.nih.gov/). AutoDock 4.2 software was used to process the protein as follows. Next, the Lamarckian genetic algorithm was chosen, then the small molecular ligands flexible bond was set to be rotatable and the receptor protein was set to a semiflexible butt joint. Then run Autogrid 4 and Autodock 4 for molecular docking. Finally, visualization was performed using the PYMOL2.2.0 software.

### RNA sequencing analysis

U251 cells were treated with NKT at a concentration of 200 µM for 24 h. Total RNA was then extracted using RNAiso Plus reagent (9108, Takara, Japan) and submitted to Novogene Corporation (Beijing, China) for whole RNA sequencing analysis. The quantity and integrity of the RNA samples were evaluated using the RNA Nano 6000 Assay Kit on the Bioanalyzer 2100 system (Agilent Technologies, CA, USA). Statistical significance was determined by adjusting the P-values with the Benjamini and Hochberg method to control for the false discovery rate. A threshold for significant differential expression was set at P adj ≤ 0.05 and |log2(fold change)| ≥ 1. The data generated from this analysis have been deposited in the Gene Expression Omnibus database under accession number GSE235973.

### Plasmid construction and cell transfection

*ATF4* shRNA sequences (5’-GCCTAGGTCTCTTAGATGATT-3’) and *CHAC1* shRNA sequences (5’-GAAGTACCTGAATGTGCGAGA-3’) were inserted into the PLKO.1 using restriction enzymes EcoRI and AgeI. Successfully constructed plasmids were transfected into U251 cells when the cell density reached 70–80% for 24–72 h before being detected by G418. Dosing screening was carried out at a starting concentration of 100 g/mL G418 until all cells fluoresced. The cell line that successfully knocked down the gene was chosen for the following tests.

### Cell viability assay

The CCK-8 cell counting kit (A311-01/02, Vazyme Biotech, Nanjing, China) was used to measure cell viability. In 96-well plates, cells were planted at a density of 1000 cells per well. After attachment, the cells were starved for 24 h before being treated with gradient concentrations of NKT (100 µM, 200 µM, 300 µM) and cultivated for different lengths of time (between 24 and 48 h). Each well was then added with 10 µL of CCK-8 solution before being incubated for 2 h at 37 °C. By using a microplate reader (Varioskan Flash, Thermo scientific, USA), the optical density of cells was assessed at a wavelength of 450 nm.

### Colony formation analysis

500 C6 cells and U251 cells were seeded onto 6-well plates. After attachment, the cells were treated with 200 µM NKT. The cells were then grown for 7 days at 37 °C in a 5% CO_2_ incubator. The cells were first rinsed with 0.01 M PBS, then fixed for 30 min with 4% paraformaldehyde (PFA), stained for 20 min with 0.1% crystal violet solution, and then washed three times with 0.01 M PBS. Using a scanner (HP Laser MFP 131 133 135–138), images were gathered.

### Transwell and wound-healing assays

For the transwell migration assay, 25,000 /mL cells were suspended in the upper chamber of a 24-well transwell plate (Corning Costar, Corning, NY). 200 µL of serum-free medium with 200 µM NKT was added to the upper chamber and 500 µL of medium containing 10% serum was added to the lower space. After 24 h of coculture, the cells were fixed with 4% PFA for 20 min, then stained for 20 min with 0.1% crystal violet solution and counted under a Nikon light microscope (Model Eclipse Ci-S; Nikon Corporation, Tokyo, Japan). For the wound-healing assay, 80,000/mL cells were cultured in 24-well plates to achieve 95% confluence. A sterile plastic tip (200 µL) was used to scratch and then monolayer cells were cultured in a serum-free medium. Images were taken to document the wound at certain time intervals (0 h, 24 h, and 48 h). With the use of ImageJ, the wound area and healing rate were determined.

### Flow cytometric analysis

To investigate nootkatone’s impact on cell death and the cell cycle, flow cytometry was performed. The Annexin V-FITC/PI Apoptosis Detection Kit (#A00947, MULTI SCIENCES, Hangzhou, China) was used to assess cell death. The Cell Cycle Staining Kit (#A01031, MULTI SCIENCES, Hangzhou, China) was used to measure the cell cycle. After receiving medication (NKT with 200 µM, 24 h) treatment, all flow operations adhered to the manufacturer’s recommendations. By using a CytoFlex S (Beckman, USA), the instructions were followed to complete the cell apoptosis and cycle analysis.

### Western blotting

Drug-treated (NKT with 200 µM, 24 h) cells and processed tissues were lysed by RIPA lysis buffer (R0010, Solarbio, Beijing, China), and left on ice for 30 min. Following centrifugation at 1, 2000 g for 20 min, protein was measured using the BCA quantification kit (23225, Thermo Scientific), extracted with 5 × loading buffer, and denatured at 95 °C for 8 min. Then the protein samples were separated using 10% sodium dodecyl sulphate-polyacrylamide gel electrophoresis (SDS-PAGE) and were transferred onto PVDF membranes. After blocking with 5% milk for 1 h at RT, the immunoblots were incubated with following primary antibodies at 4 ℃ overnight: rabbit anti-Cyclin D1 (#55506, CST, 1:1000), rabbit anti-N-Cadherin (#ET1607-37, Huabio, 1:1000), rabbit anti- Cleaved-caspase3 (#ab2302, Abcam, 1:500), rabbit anti-GPX4 (#52455S, CST, 1:1000), rabbit anti-ATF4 (#ET1612-37, Huabio, 1:1000), rabbit anti-DDIT3 (CHOP) (#ET1703-05, Huabio, 1:1000), rabbit anti-CHAC1 (#ER1906-19, Huabio, 1:1000), mouse anti-β-actin (#200068–8F10, ZEN BIO, 1:5000), mouse anti-GAPDH (#200306-7E4, ZEN BIO, 1:5000), mouse anti-Tubulin (#200608, ZEN BIO, 1:5000). Membranes were then incubated with the horseradish peroxidase (HRP) conjugated secondary antibodies [1:10000, Sigma, A0545 (goat anti-rabbit), A9044 (goat anti-mouse)] for 50 min at RT, detected by the BLT GelView 6000Plus (Guangzhou Biolight Biotechnology Co., Ltd., China) with applying the FDbio-Dura ECL luminescent liquid (FD8020, Fdbio science) and analyzed by using ImageJ.

### Immunofluorescence analysis

Drug-treated (NKT with 200 µM, 24 h) cells were washed once with 0.01 M PBS, and fixed in 4% PFA for 25 min. Then, the fixed cells were permeabilized with 0.1% Triton X-100 PBS solution for 15 min, and blocked with 5% Bovine Serum Albumin (BSA) (V900933, Sigma-Aldrich) solution at RT for 50 min. Subsequently, cells were incubated with primary antibodies (mouse anti-PH3, ab14955, Abcam, 1:2500) at 4 ℃ overnight, washed with 0.01 M PBS three times for 5 min each, and then incubated with fluorescent secondary antibodies (Alexa Fluor™ 546 goat anti-mouse IgG (H + L) (A11030, Invitrogen, 1:1000) at RT for 1 h. Olympus SLIDEVIEW™ VS200 microscope (Olympus, Japan) or Olympus confocal system (FV3000) (Olympus, Japan) was used to acquire images, and Adobe Photoshop CS 10.0 software was used to process the illustrative diagram.

### Orthotopic transplantation mouse model

The Animal Ethical and Welfare Committee at Hangzhou Normal University examined and approved the animal study. A uniform suspension of GL261 cells (100,000 cells in 1 µL of 0.01 M PBS) was implanted into the right striatum (+ 0.5 mm A/P, − 1.75 mm M/L, and − 4.0 mm D/V) with a 69,100 rotary digital stereotaxic apparatus (RWD, Shenzhen, China) to build the intracranial GBM model. After 7 days of tumor transplantation, the mice were randomly divided into two groups (8 mice in one group): Control group and Nootkatone group. Mice were administered 40 mg/kg nootkatone or equivalent saline (containing 2.5% DMSO) daily through intraperitoneal injection. After two weeks of administration, the bioluminescence imaging of tumors was acquired and analyzed by a 3D imaging system in small animals (PhotonIMAGER Optima, Biospace Lab, France). All mice were sacrificed 21 days after tumor transplantation. We repeated the same procedure to assess the impact of NKT on the survival time of mice, but instead of sacrificing them 21 days after tumor implantation, we waited for them to die naturally.

### **Measurement of ROS**

According to the reactive oxygen species test kit (C3890, APExBIO, Shanghai, China), the redox-sensitive fluorescent probe dichloro-dihydro-fluorescein diacetate (DCFH-DA) was employed to analyze intracellular ROS. Following a 30-min incubation period at 37 °C in the presence of DCFH-DA (1:5,000), cells treated with 200 µM NKT for 24 h were immediately subjected to flow cytometric analysis (CytoFLEX S, Beckman Coulter).

### Hematoxylin-eosin (HE) staining

After two weeks of administration, the mice were anesthetized and subjected to cardiac perfusion to rule out the influence of blood on subsequent experiments. The tissue was placed in 4% PFA for 4 h and then transferred to 30% sucrose solution for dehydration. After complete settlement, the tissues were embedded in OCT (optimal cutting temperature compound, SAKURA, 4583) for the frozen section. The brains (20 μm) were stained in hematoxylin solution, cleaned with ultrapure water, and then stained with eosin solution. The sections were dehydrated using ethanol in three different concentrations (75%, 95%, and 100%). After that, xylene was used to make the sections translucent. At last, neutral resin was used to seal the tissues.

### PI staining

The living cells treated with 200 µM NKT for 24 h were washed with 0.01 M PBS, and then processed with the working solution (0.01 µg/µL) in the PI staining kit (instructions Propidium Iodide, Beyotime, ST511). After 15 min incubation in the dark, cells were observed and photographed by using an inverted fluorescent microscope (Olympus, Japan).

### RNA extraction and quantitative real-time PCR (qRT-PCR)

Total RNA was extracted by RNA-easy Isolation Reagent (R701-01, Vazyme, China) according to the manufacturer’s protocol. And the extracted RNA was quantified with Thermo Scientific™ NanoDrop™ One (840-317400, Thermo Scientific, the USA). cDNA synthesis was performed with the cDNA DyNAmo Kit (R211–01/ 02, Vazyme, China). qPCR was performed with the SYBR Green PCR master mix (Q511–02/03, Vazyme, Nanjing, China). qPCR cycling conditions were programmed as such: pre-denaturation step (95 ℃, 5 min), cycling step (denaturation at 94 ℃, 15 s; annealing at 55 ℃, 30 s; extension for 72 ℃, 30 s × 40 cycles), finally using a melting curve analysis to confirm the specificity of the PCR products. The C_T_ value for each group of samples were corrected by the corresponding β-actin C_T_ values. Gene expression levels were expressed as ΔC_T_ = C_T_ gene - Ct reference, and fold changes were calculated by the 2^−ΔΔCT^ method. The primers used in this study were synthesized by Zhejiang Tsingke and presented as follows: *ATF4*, 5’-GTTCTCCAGCGACAAGGCTA-3’ and 5’- CGGAGAAGGCATCCTCCTTG-3’; *CHOP* 5’-GGAAACAGAGTGGTCATTCCC-3’ and 5’-CTGCTTGAGCCGTTCAT-TCTC-3’; *CHAC1* 5’- AGTGCAAGGGGAGCAGAACC-3’ and 5’-TGCCAGACGC-AGCAAGTATT-3’ β-actin, 5’-AGACTTCGAGCAGGA-GATGGC-3’ and 5’-TCGT-TGCCAATAGTGATGACCTG-3’.

### Statistical analysis

The analysis was performed using GraphPad Prism software version 8.0 (Graph Pad Software, Inc., La Jolla, CA, USA). The statistical significance between different groups was assessed by student’s *t*-test, one-way ANOVA, two-way ANOVA, two-way RM ANOVA (Tukey’s test, Bonferroni’s test, or Sidak’s test was used for post hoc comparison) or the Kruskal-Wallis test (Dunn’s Multiple Comparison Test was used for post hoc comparison) and the Kaplan–Meier method. All experiments were repeated at least three times and presented as the mean ± SEM. *P* < 0.05 was considered to indicate a statistically significant difference.

## Results and discussion

### Nootkatone inhibited the proliferation and growth of GBM cells in vitro

**Fig. 1:**
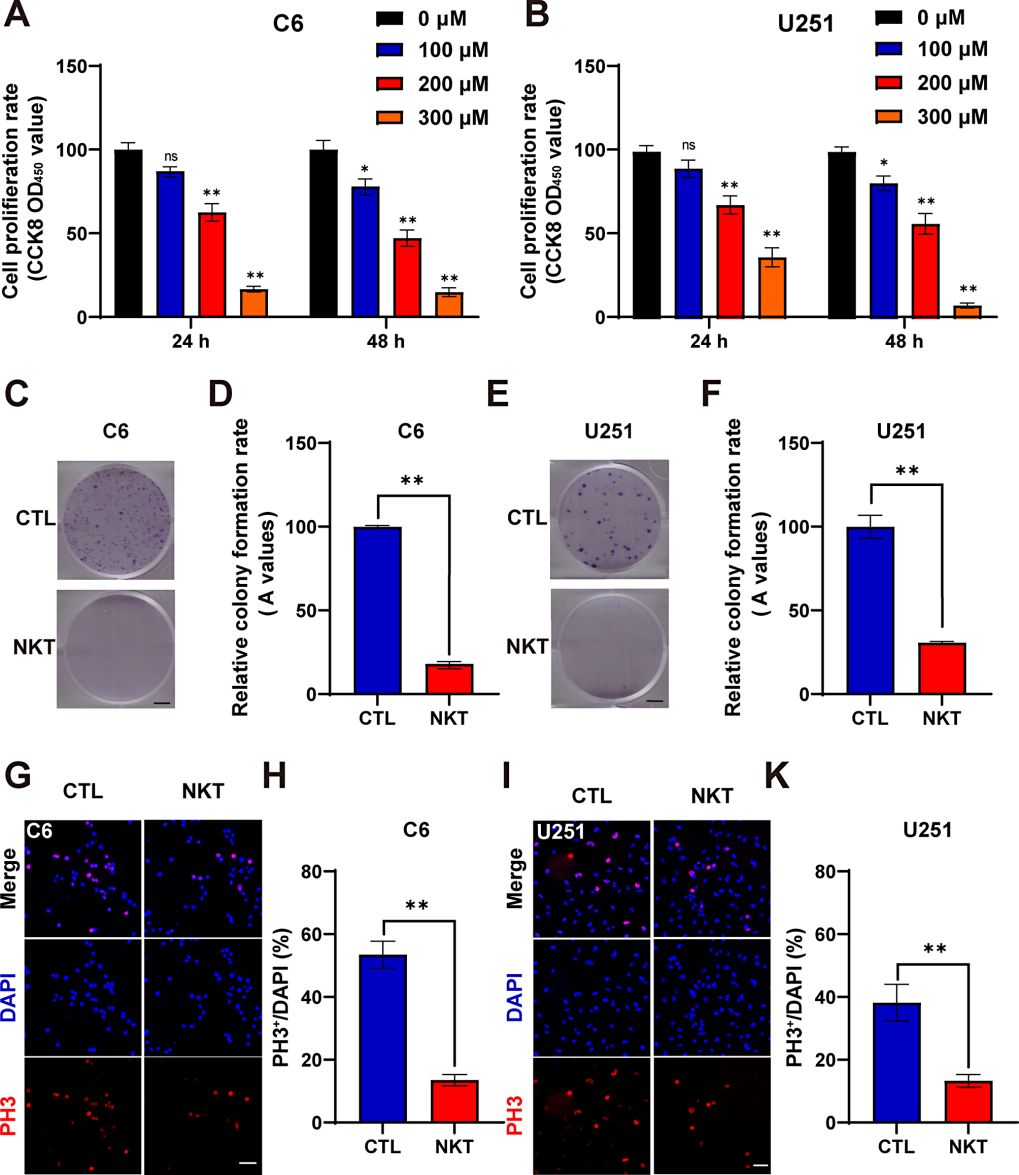
NKT inhibited the proliferation and growth of GBM cells in vitro. (**A**, **B**) The cell viability of C6 cells and U251 cells treated with gradient concentrations (0 µM, 100 µM, 200 µM, 300 µM) of nootkatone detected by CCK8 assays (*n* = 5/group, one-way ANOVA, Tukey’s test was performed for the multiple comparison). (**C**, **E**) Cell colony formation of C6 cells and U251 cells treated with DMSO or NKT (200 µM) for approximately 7 days. Scale bar, 500 μm. (**D**, **F**) Quantitative analysis of colony number of C6 cells (**D**) and U251 cells (**F**) (the surviving colonies > 10 cells) as shown in (**C**, **E**) (*n* = 3/group, *t*-test). (**G**, **I**) Representative images of PH3 staining (red) in C6 cells and U251 cells after being treated with DMSO or 200 µM nootkatone for 48 h. Scale bars, 50 μm. (**H**, **K**) Quantitative analysis of the percentages of PH3^+^ cells over total C6 cells and U251 cells in one field as shown in (**G**, **I**) (*n* = 5/group, *t*-test). Data were mean ± SEM. ^*^P < 0.05, ^**^P < 0.01

We initially assessed whether NKT affected the growth of C6 and U251 cells (two GBM cell lines) in vitro. As shown in Fig. 1A-B, CCK8 assays demonstrated that NKT dramatically reduced the viability of C6 cells and U251 cells in dose- and time-dependent manners. Besides, the CCK-8 assay was also performed on mouse primary astrocytes and the results showed that NKT was not significantly toxic to normal cells at 24 h, but it was mildly toxic to normal cells at 48 h (Figure S1). Furthermore, the result of a colony-formation assay further confirmed that NKT significantly inhibited the proliferation and growth of GBM cells (Fig. 1C-F). Additionally, to further ascertain the anti-proliferation effects of NKT on GBM cells, immunostaining results revealed that, in a dose-dependent manner, NKT treatment significantly decreased the proportion of PH3^+^ (a marker of mitotic cells) cells in C6 and U251 cells (Fig. 1G-J). Together, our findings indicated that NKT restricted the proliferation and growth of GBM cells in vitro.

### Nootkatone suppressed the migration of GBM cells

**Fig. 2:**
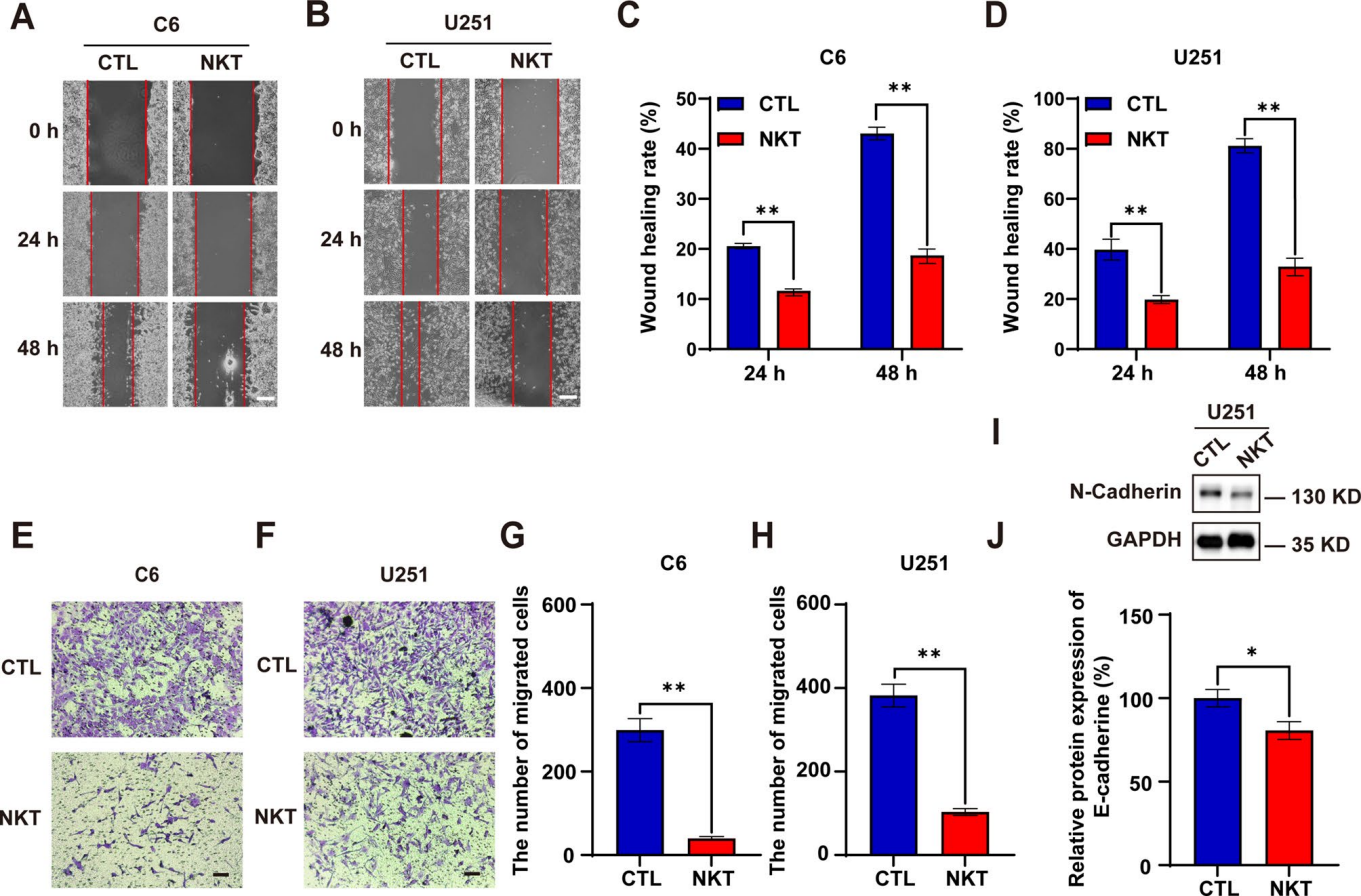
NKT suppressed the migration of GBM cells. (**A**, **B**) Representative images of C6 cells and U251 cells treated with 200 µM NKT in wound healing assays. Phase-contrast images were acquired at 0, 24 and 48 h after scratching. Scale bars, 200 μm. (**C**, **D**) Quantitative analysis of the wound healing area of C6 cells and U251 cells treated with NKT as shown in (**A-B**) (*n* = 12/group, two-way ANOVA, Bonferroni’s test was performed for the multiple comparison). (**E**, **F**) Representative images of C6 cells and U251 cells treated with 200 µM NKT for 24 h in transwell migration assay. Scale bar, 200 μm. (**G**, **H**) Quantitative analysis of the numbers of migrated C6 cells and U251 (*n* = 12/group, *t*-test) cells counted in representative high-power fields per transwell plate. (**I**) The expression of N-cadherin in U251 cells was detected after being treated with 200 µM NKT for 48 h by western blot. (**J**) Quantification analysis of the relative N-cadherin in U251 cells (normalized to β-actin, *n* = 6/group, Mann-Whitney test) as shown in (**I**). Data were mean ± SEM. ^*^P < 0.05, ^**^P < 0.01

To evaluate the effects of NKT on the migration ability of GBM cells, wound healing assay was carried out. After 48 h of NKT incubation, the results showed that NKT greatly reduced the migration of C6 and U251 cells (Fig. 2A-D). A transwell experiment was subsequently employed to validate NKT’s inhibitory effect on invasive cell migration. Besides, the results of transwell experiments using NKT at different concentrations showed that as the concentration increased, the number of U251 cells passing through the chamber gradually decreased, indicating that NKT can inhibit the migration of U251 cells in a concentration dependent manner (Figure S2A-B). As shown in Fig. 2E-H, NKT significantly suppressed the migration of C6 cells and U251 cells after exposure for 24 h. Additionally, we discovered that NKT-treated U251 cells had lower expressed levels of the migration-associated protein N-cadherin (Fig. 2I-J). Taken together, these results strongly suggested that NKT inhibited the migration of GBM cells.

### Nootkatone arrested cell cycle at G2/M phase in GBM cells

**Fig. 3:**
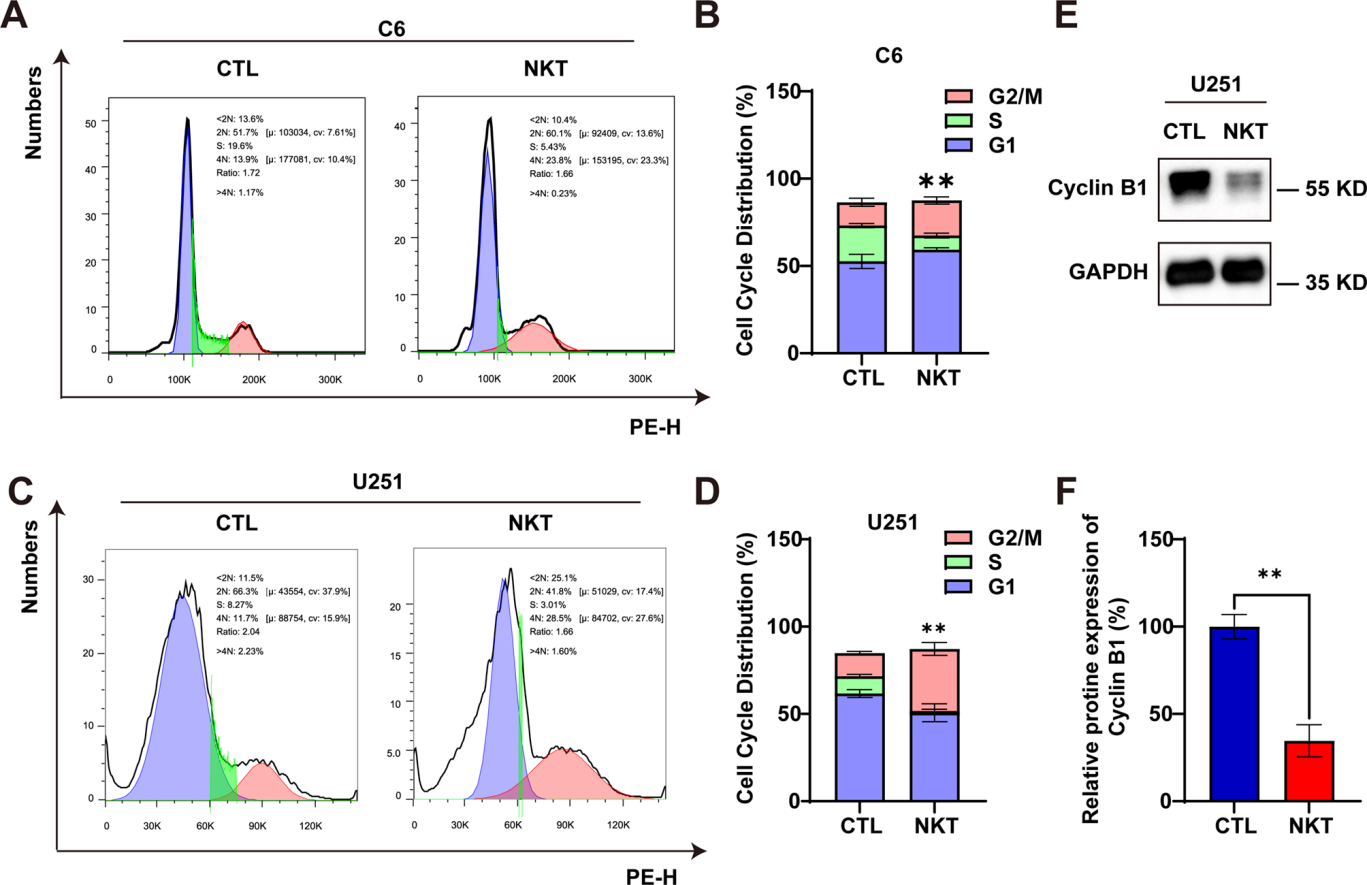
NKT arrested cell cycle of GBM cells at G2/M phase. (**A-D**) Representative flow cytometry results of cell cycle (**A**, **C**) and quantitative analysis in C6 cells and U251 cells after NKT treatment for 48 h (**B**, **D**, *n* = 5/group, *t*-test). (**E**) The expression of Cyclin B1 in C6 cells and U251 cells treated with NKT for 48 h. (**F**) Quantification analysis of the relative Cyclin B1 level in U251 cells as shown in (**E**) (normalized to GAPDH, *n* = 6/group, Mann-Whitney test). Data were mean ± SEM. ^**^P < 0.01

To further confirm whether NKT could inhibit cell proliferation by inducing cell cycle arrest, flow cytometry was performed. The results illustrated that NKT treatment induced G2/M phase arrest in C6 cells and U251 cells together with a reduction in the quantity of cells in the G1 and S phases (Fig. 3A-D). Furthermore, western blot analysis showed that the expression level of Cyclin B1 (Cell cycle regulatory protein that plays vital roles in the G2/M phase) was obviously decreased in NKT-treated U251 cells (Fig. 3E, F). Taken together, these results suggested that NKT induced G2/M phase arrest in GBM cells.

### Nootkatone triggered the cell death of GBM cells

**Fig. 4:**
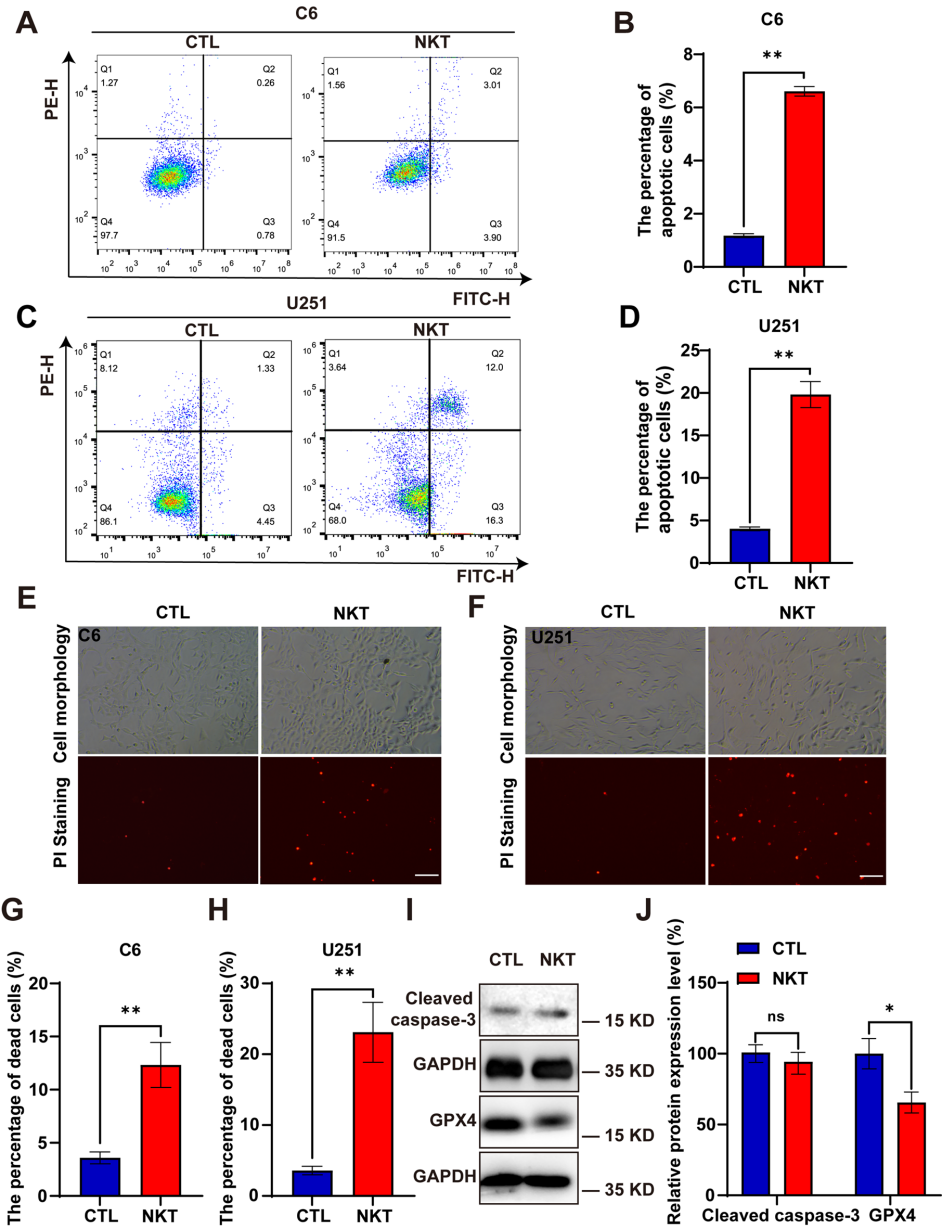
NKT induced the cell death of GBM cells. (**A**, **C**) Representative results of annexin V/FITC/PI staining in C6 cells and U251 cells treated with NKT for 48 h in flow cytometry assays. (**B**, **D**) Quantitative analysis of the percentages of dead cells in C6 cells and U251 cells (*n* = 5/group, *t*-test). (**E**, **F**) Representative images of PI staining in C6 cells and U251 cells after being treated with DMSO or 200 µM NKT for 48 h. Scale bars, 200 μm. (**G**, **H**) Quantitative analysis of the percentage of PI^+^ cells in one field as shown in (**E**) and (**F**) (*n* = 6/group, *t*-test). (**I**) The expression of Cleaved-caspase3 and GPX4 in U251 cells treated with NKT for 48 h. (**J**) Quantification analysis of the relative Cleaved-caspase3 and GPX4 level in U251 cells as shown in (**I**) (normalized to GAPDH, *n* = 6/group, Mann-Whitney test). Data were mean ± SEM. ^**^P < 0.01

Next, we examined the effect of NKT on cell death by using flow cytometry assays and PI (Propidium iodide) staining. We calculated the ratio of PI + cells in the fluorescence field to the total number of cells in the brightfield, then plotted this ratio on a histogram. As shown in Fig. 4A-D, the percentages of dead cells were significantly increased by approximately 6% and 22%, respectively, in C6 cells and U251 cells treated with NKT for 48 h. PI staining further suggested that the percentages of PI^+^ cells were increased by approximately 10% and 20%, respectively, in C6 cells and U251 cells treated with NKT for 48 h (Fig. 4E-H). Furthermore, western blot analysis showed that the expression level of Cleaved-caspase3 (A key executor of apoptosis) had not changed (Fig. 4I-J). Moreover, the expression level of GPX4 (An intracellular selenoprotein antioxidant enzyme that is an important regulator of ferroptosis) obviously decreased in NKT-treated U251 cells (Fig. 4I-J). Taken together, these results suggested that NKT treatment triggered the death of GBM cells.

### Nootkatone activated the ATF4/ CHOP/CHAC1 pathway in GBM cells

We next examined the mechanism of anti-GBM by NKT. After U251 cells were treated with 200 µM NKT for 24 h, RNA was extracted, and the transcripts were sequenced. Differentially expressed genes were screened in NKT-treated U251 cells, and results showed that 1,189 mRNA and long non-coding RNA (lncRNA) were upregulated and 1,526 mRNA and lncRNA were downregulated in NKT-treated U251 cells (GSE235973). Interestingly, we found that the expression of *ATF4* (activating transcription factor 4), *CHOP* (C/EBP homology protein) and *CHAC1* (ChaC glutathione specific gamma-glutamylcyclotransferase 1) were significantly upregulated. Furthermore, we verified that NKT significantly increases the expression of ATF4 in a dose-dependent manner through western blotting (Figure S2C-D).

**Fig. 5:**
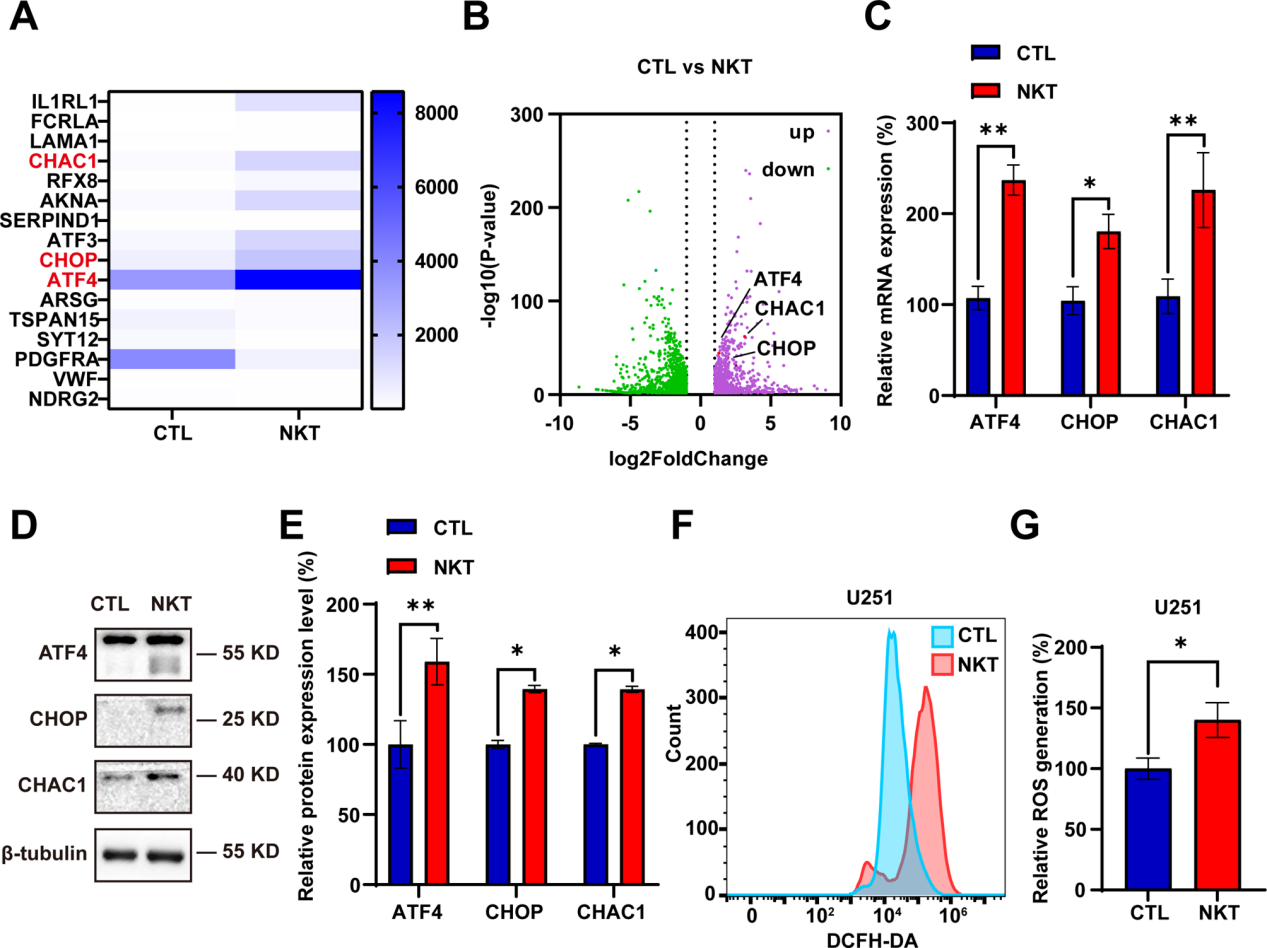
NKT activated the ATF4-CHOP-CHAC1 pathway in GBM cells. (**A**) A representative heat map of the upregulated and downregulated genes in NKT-treated U251 cells compared with the control. The depth of blue represents the level of gene expression. The expression of several genes such as *ATF4* and *CHOP* increased significantly. (**B**) The volcano figure shows the variation in 200 µM NKT-treated U251 cells and the control-treated U251 cells. The purple plot indicates RNAs with greater than 2.0-fold upregulation between the two groups, while the green plot indicates the downregulated RNAs. (**C**) The mRNA expression of *ATF4*, *CHOP*, *CHAC1* in U251 cells was detected after being treated with 200 µM NKT for 48 h by qPCR (*n* = 8/group, *t*-test). (**E**) The expression of ATF4, CHOP, CHAC1 in U251 cells was detected after being treated with 200 µM NKT for 48 h by western blot. (**F**) Quantification analysis of relative ATF4, CHOP, CHAC1 (normalized to β-tubulin, *n* = 3/group, *t*-test) in U251 cells as shown in (**E**). (**G**) Representative results of ROS analysis in NKT-treated U251. (**H**) Quantitative analysis of ROS in U251 cells (*n* = 4/group, *t*-test) as shown in (**G**). Data were mean ± SEM. ^*^P < 0.05, ^**^P < 0.01

ROS induces endoplasmic reticulum stress (ERS) and promotes unfolded protein response (UPR) (Kim et al. 2017; Su et al. 2019). However, when ERS is prolonged and severe, UPR promotes programmed cell death via phosphorylation of eukaryotic initiation factor-2α (P-eIF2α) (Seervi et al. 2018). P-eIF2α selectively enhances the expression of ATF4 and promotes downstream protein expression (CHOP and CHAC1) (Hiramatsu et al. 2014). CHAC1 has γ-glutamylcyclotransferase activity and degrades glutathione, which induces cell death (Mungrue et al. 2009). The pathway that tandem with these three genes has been shown to play a key role in inhibiting the growth of several tumors (Wang et al. 2019, 2022a, b). Real-time quantitative PCR (Fig. 5C) and western blot (Fig. 5D-E) further confirmed that ATF4-CHOP-CHAC1 pathway was activated in NKT-treated U251 cells in terms of genes and proteins, respectively. Given this pathway could lead to ROS production increased (Wang et al. 2019), we then analyzed the intracellular ROS and found it was indeed increased (Fig. 5F-G).

Homogenous molecular docking was used to confirm the binding site between NKT and the upstream-gene ATF4. The binding site of ATF4 to NKT was shown in Figure S3A-B using Autodock 4. Furthermore, the GEPIA database showed that expression of downstream-gene CHAC1 in this signaling pathway was much lower in tumor tissues than in normal tissues, which meant activating this gene has therapeutic implications for GBM (Figure S3C-D).

Taken together, these results suggested that NKT activated the ATF4-CHOP-CHAC1 pathway in GBM cells.

### Nootkatone inhibited the proliferation and migration of GBM cells by upregulation of ATF4-CHOP-CHAC1 signaling pathway

**Fig. 6:**
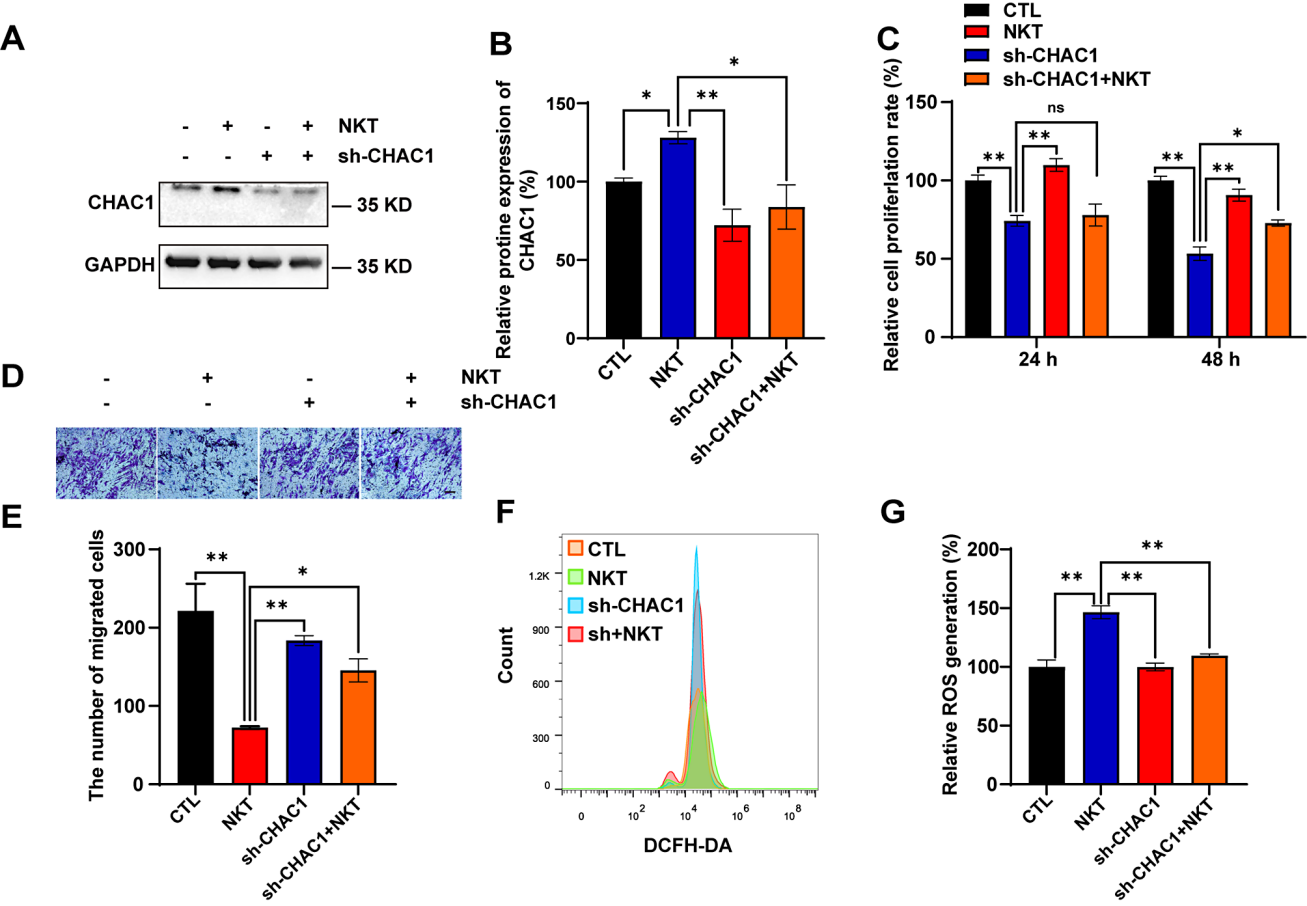
NKT inhibited the tumorigenesis of GBM by upregulation of activating ATF4-CHOP-CHAC1. (**A**) The expression of CHAC1 in U251 cells transfected with sh-CHAC1 constructs and then treated with 200 µM NKT for 48 h. (**B**) Quantification analysis of the relative CHAC1 level in U251 cells as shown in (**A**) (normalized to β-tubulin, *n* = 6/group, one-way ANOVA, Tukey’s test was performed for the multiple comparison). (**C**) The effects of sh-CHAC1 on the viability of U251 cells as detected by CCK8 assays after 24 h and 48 h of 200 µM NKT treatment (*n* = 5/group, one-way ANOVA, Tukey’s test was performed for the multiple comparison). (**D**) Typical images of U251 cells transfected with sh-CHAC1 constructs and then treated with 200 µM NKT for 24 h in transwell migration assay. Scale bar, 200 μm. (**E**) The numbers of migrated cells were counted in representative high-power fields per transwell plate as shown in (**D**) (*n* = 5/group, one-way ANOVA, Tukey’s test was performed for the multiple comparison). (**F**) Representative results of ROS analysis in NKT-treated U251 cell. (**G**) Quantitative analysis of ROS in U251 cells (*n* = 4/group, *t*-test) as shown in (**F**). Data were mean ± SEM. ^*^P < 0.05, ^**^P < 0.01

An RNAi plasmid for down-regulating *ATF4* and *CHAC1* was constructed in order to further verify the role of activating the ATF4-CHOP-CHAC1 pathway in the regulation of GBM tumorigenesis by NKT. As shown in Figure S4A-B, *ATF4* was indeed knocked down in U251 cells. Interestingly, NKT-induced cell viability reduction, proliferation inhibition (Figure S4C), and migratory inhibition (Figure S4D-E) in U251 cells were partially recovered by knockdown of *ATF4* (*sh-ATF4*). Meanwhile, following the knockdown of CHAC1 (sh-CHAC1), the reduced viability and inhibited proliferation of U251 cells induced by NKT after 48 h were partially restored (Fig. 6C). A similar change was observed in the migratory inhibition (Fig. 6D-E), and increased ROS production (Fig. 6F-G). Taken together, these findings provided compelling evidence that NKT inhibited the proliferation and migration of GBM cells by activating ATF4-CHOP-CHAC1 pathway.

### Nootkatone inhibited GBM tumor growth in vivo through activating ATF4-CHOP-CHAC1 pathway

**Fig. 7:**
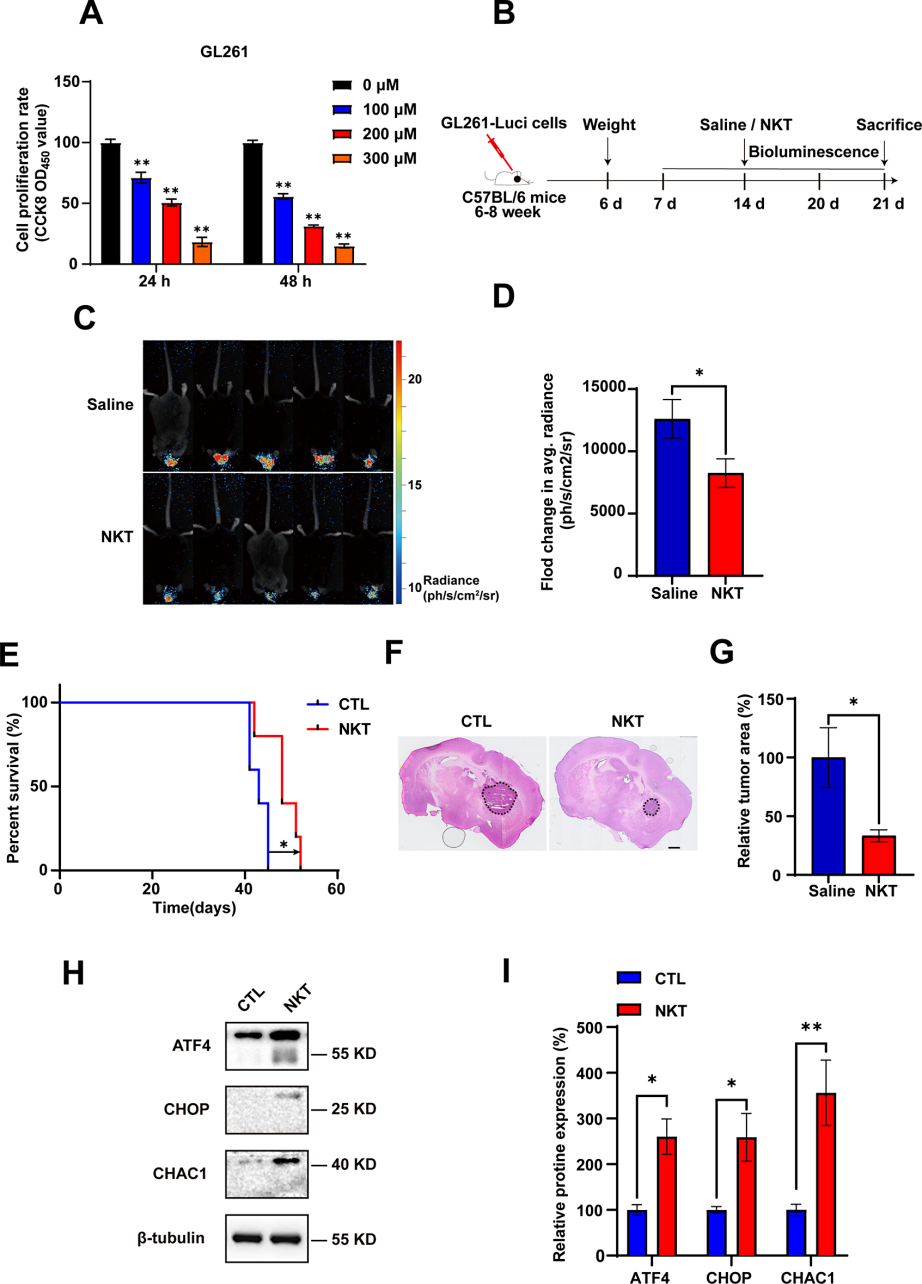
NKT inhibited GBM growth in vivo through activating ATF4-CHOP-CHAC1 pathway. (**A**) The cell viability of GL261 cells treated with gradient concentrations (0 µM, 100 µM, 200 µM, 300 µM) of NKT detected by CCK8 assays (*n* = 5/group, one-way ANOVA, Tukey’s test was performed for the multiple comparison). (**B**) Scheme of orthotopic GBM mice model experiment design. Intracranial tumors were established by stereotactically implanting GL261 cells. When the tumors grew to 7 days, the tumor-planted male C57BL/6 mice were separated into two groups randomly. And the equal parts of saline (containing 2.5% DMSO) or NKT were administrated each day. When NKT given for two weeks, the mice were executed. (**C**) Bioluminescent imaging of disseminated GL261-Luc orthotopic C57BL/6 mice posttreatment with NKT and saline. (**D**) Fold change in average radiance per mouse at experimental endpoint (day 21) was analyzed for each treatment group (*n* = 5/group, *t*-test). (**E**) Survival curve. The administration of 40 mg/kg NKT extends the survival duration of mice (*n* = 5/group). (**F**) Representative images of HE staining of whole-brain sections from saline and NKT groups. Scale bar = 1 mm. (**G**) Quantification analysis of the area of GBM in (**F**) (*n* = 3/group, *t*-test). (**H**) The expression of ATF4, CHOP and CHAC1 in orthotopic tumor tissue was detected by western blot. (**I**) Quantification analysis of ATF4, CHOP and CHAC1 levels as shown in (**H**) (normalized to β-tubulin, *n* = 3/group, *t*-test). Data were mean ± SEM. ^*^P < 0.05, ^**^P < 0.01

In view of the obvious anti-GBM activity of NKT in vitro, we next examined whether NKT could inhibit the growth of intracranial GBM in vivo with the murine orthotopic GL261 cells. Firstly, the result of CCK-8 assay on GL261 cells, which is syngeneic on the C57BL/6 mice background, indicated that NKT could obviously suppress the cell viability in a concentration dependent manner (Fig. 7A). Next, the striatum of C57BL/6 mice was injected with GL261 cells carrying the luciferase reporter gene using a brain stereotaxic apparatus. The mice were randomly divided into two experimental groups after 7 days of modeling: the control group, which received saline containing 2.5% DMSO, and the NKT group, which received 40 mg/kg of NKT. Mice were administered with NKT intraperitoneally once a day.

After 14 days of administration, the size of the tumor was observed by a three-dimensional bioluminescence imaging system in small animals (Fig. 7B). According to the bioluminescence fold change of GL261-luciferase cells in C57BL/6 mice brains, we found that the tumor volume in the NKT group was significantly decreased, compared with that in the control group, (Fig. 7C-D). The survival rate analysis indicated that mice treated with 40 mg/kg of NKT exhibited a significantly extended lifespan compared to the control group (Fig. 7E). Similar results were shown for statistics of tumor area after HE staining (Fig. 7F-G). Furthermore, ATF4, CHOP and CHAC1 in the NKT group were significantly upregulated in the orthotopic model mice (Fig. 7H-I). Taken together, these results suggested that NKT inhibited GBM growth in vivo through activating ATF4-CHOP-CHAC1 pathway.

## Discussion

The present study aimed to investigate the antitumor effects of NKT on GBM cells, and the results showed that NKT inhibited the proliferation and migration of GBM cells, and induced GBM cell death by increasing the production of ROS in dose- and time-dependent manners. Furthermore, NKT activated the ATF4-CHOP-CHAC1 signaling pathway to inhibit GBM growth both in vitro and in vivo. These findings suggest that NKT has the potential to be a treatment option for GBM.

*Alpinia oxyphylla Miq*, a traditional Chinese medicine, has long been used to treat various conditions (Xu et al. 2020) including diarrhea (Wang et al. 2015), dementia (Liu et al. 2014), inflammation (An et al. 2006), cancer and other diseases (Zhang et al. 2018). In 1984, the corresponding component alcohol nootkatol was first isolated from this important medicinal plant *Alpinia oxyphylla Miq* (Shoji et al. 1984). Among the many active ingredients, NKT is one of the most abundant ones (Nemmar et al. 2018). Recent pharmacological studies have shown that NKT has a great effect on improving cognitive impairment and alleviating pathological brain injury characterized by neurodegeneration (Wang et al. 2018; He et al. 2019). NKT has also been reported to significantly affect the progression of non-small-cell lung carcinoma (Hung et al. 2019) and colorectal cancer (Yoo et al. 2020). Consistent with these previous studies, our results showed that NKT inhibited the proliferation and growth of GBM cells in vitro and in vivo, along with the inhibition of cell migration.

To elucidate the underlying mechanism of the inhibition of NKT on GBM cells, the transcriptional sequencing was screened, and the results suggested that NKT upregulated ERS (endoplasmic reticulum stress)-related genes (*ATF4*, *CHOP*, *CHAC1*) in NKT-treated GBM cells. Furthermore, and these genes were confirmed to be upregulated in NKT-treated GBM cells by qPCR and western blot.

ROS-induced cell death is considered a promising anti-tumor method recently (Wang et al. 2022a, b; Kirtonia et al. 2020). ROS are a series of molecules produced by oxidative metabolism in cells, including singlet oxygen (primary excited state), superoxide anion (single electron state), hydroxyl radical (three electron state) and hydrogen peroxide (two electron reduction state) (Jia et al. 2020; Poillet-Perez et al. 2015). Notably, at low levels, ROS can act as important signaling molecules involved in multiple regulation of biological and physiological processes (Yu et al. 2022; Chen et al. 2020a, b). In the tumor microenvironment, low levels of ROS play an important role in signaling and angiogenesis, which can promote tumor cell proliferation and metastasis (Song et al. 2023; Chen et al. 2020a, b). In contrast, high levels of ROS can damage the DNA of tumor cells, leading to some degree of tumor cell death (Kim et al. 2016; Mishra et al. 2019). In addition, most chemotherapy drugs produce ROS in cancer cells (Zhao et al. 2023; Bai et al. 2021). Anthracycline, such as doxorubicin, daunorubicin and epirubicin, can cause elevated level of ROS (Xiang et al. 2021; Lou et al. 2021). In contrast, vinca alkaloids, taxanes, nucleotide analogs, and antimetabolites, including folic acid, produce lower levels of ROS (Sahoo et al. 2022). Chen et al. identified that NKT inhibited the growth of retinoblastoma cells *via* autophagy, endogenous ROS production, cell cycle arrest and inhibition of NF-κB signaling pathway (Zhu et al. 2020). Thus, we hypothesize that NKT may affect GBM progression through increasing ROS level.

ATF4 is an important regulator of endoplasmic reticulum stress and has recently been found to be a mediator of ferroptosis. As a basic leucine zipper (Bzip) transcription factor which regulates amino acid metabolism, cellular redox states and anti-stress responses (Ye and Koumenis 2009), ATF4 has dual effects on cell death (Wortel et al. 2017). On the one hand, tumor cells often use ATF4 to reduce stress caused by rapid proliferation and nutrient restriction in the growing tumor mass (Hao et al. 2016; Ren et al. 2015; Hart et al. 2012). Overexpression of miR-1283 inhibited the proliferation and invasion of GBM cells by directly down-regulating the expression of ATF4 (Chen et al. 2019a, b). Dihydroartemisinin could enhance the anticancer activity by increasing ferroptosis in GBM through inhibiting the PERK-ATF4-HSPA5-GPX4 pathway (Chen et al. 2019a, b). On the other hand, in some cases, ATF4 expression was found to sensitize tumor cells to therapy-induced cell death (Ishizawa et al. 2016; Qing et al. 2012). Sevoflurane induces ferroptosis of GBM cells through activating the ATF4-CHAC1 pathway (Xu et al. 2022). Zhu et al. identified that NKT led to the increase of ROS production in retinoblastoma dose-dependently (Zhu et al. 2020). Ferroptosis is caused by excess ROS produced by iron ions in the mitochondria that interact with NADPH oxidase enzymes (Xie et al. 2016). In some studies, the increased expression of ATF4 has been shown to inhibit the proliferation and metastasis of GBM cells, which was consistent with our results (Sui et al. 2020a, b).

Our study confirmed a significant increase in ATF4 protein levels in U251 cells by NKT treatment. Through the rescue experiments, we demonstrated that inhibition of ATF4 attenuated NKT-mediated cell proliferation, migration, and ROS generation. Meanwhile, suppression of ATF4 obviously reversed the expression of downstream protein CHOP, CHAC1. We may speculate that NKT inhibits the proliferation of GBM cells through ATF4-mediated ferroptosis. Moreover, to confirm this pathway, we also focused on the downstream gene of this pathway. CHAC1 (a g-glutamyl cyclotransferase) causes cell ferroptosis by degrading glutathione (Wang et al. 2019). The GEPIA database showed that CHAC1 expression in tumor tissues was significantly lower than that in normal tissues. Several researches have shown that CHAC1 is regulated by ATF4 and participates in the process of cell ferroptosis. CHAC1-inhibited Notch3 signaling can influence Temozolomide-mediated cytotoxicity (Chen et al. 2017). Activation of CHAC1 is associated with the ATF4-CHOP cascade in the endoplasmic reticulum (Joo et al. 2015). Our study found that NKT activated the ATF4-CHOP-CHAC1 cascade, and suppressed the growth of GBM cells in vitro and in vivo. When the expression of ATF4 was knocked-down, the inhibition of cell growth induced by NKT was also mitigated. These results suggested that upregulation of ATF4-CHOP-CHAC1 impaired the ability of GBM cells to resist death.

## Conclusion

In conclusion, our results reveal that NKT induces GBM cell death by activating the ATF4-CHOP-CHAC1 pathway, which partially inhibits the growth and migration of GBM cells. These findings provide a new mechanism for NKT-induced anti-tumor effects in GBM cells and highlight NKT could become a potential treatment for GBM. Moreover, our study also presents a novel chemical entity with pharmacological potential, providing a promising direction for the GBM treatment with traditional Chinese medicine.

## Electronic supplementary material

Below is the link to the electronic supplementary material.

Supplementary Material 1

## Data Availability

The datasets used and/or analysed during the current study are available from the corresponding author upon reasonable request.
