# Supplementary material for: Nootkatone inhibits the progression of glioblastoma by activating the ATF4-CHOP-CHAC1 pathway
Source: Mol Med. 2025 Jan 16;31:13. doi: 10.1186/s10020-025-01064-1 (PMC11737244; doi:10.1186/s10020-025-01064-1)
Supplement: Supplementary file 1 — Supplementary Material 1 [file 10020_2025_1064_MOESM1_ESM.docx]

**Figure S1.** (**A**) The cell viability of mouse primary astrocytes with 200 μM concentrations of nootkatone in 24 h and 48 h detected by CCK8 assays (n = 5/group, one-way ANOVA, Tukey’s test was performed for the multiple comparison).

**Figure S2.** (**A**) The expression of ATF4 in U251 cells was detected after being treated with gradient concentrations (0 μM, 100 μM, 200 μM, 300 μM) of NKT for 24 h by western blot. (**B**) Quantification analysis of ATF4 (normalized to GAPDH, n = 5/group, t-test) in U251 cells as shown in (**A**). (**C**) Representative images of U251 cells treated with gradient concentration of nootkatone for 24 h in transwell migration assay. Scale bar, 200 µm. (**D**) Quantitative analysis of the numbers of migrated U251 cells counted in representative high-power fields per transwell plate (n = 12/group, *t*-test).

**Figure S3.** (**A**) 2D diagram of the predicted interactions between NKT and ATF4; backbone of the NKT is colored in grey. Ligand-protein interactions are colored depending on their type: alkyl is colored in pink, conventional hydrogen bonds are colored in green. (**B**) 3D docking mode between NKT and ATF4 based on Autodock assimilation and active site-amino acids (ARG 300). (**C-D**) Differential expression of CHAC1 in 207 normal samples and 163 GBM samples. Data were analyzed using GEPIA database (http://gepia.cancer-pku.cn/). Data were mean ± SEM. *^*^P < 0.05*.

**Figure S4.** (**A**) The expression of ATF4 in U251 cells transfected with sh-ATF4 constructs and then treated with 200 μM nootkatone for 48 h. (**B**) Quantification analysis of the relative ATF4 level in U251 cells as shown in (**A**) (normalized to β-tubulin, n = 6/group, one-way ANOVA, Tukey’s test was performed for the multiple comparison). (**C**) The effects of sh-ATF4 on the viability of U251 cells as detected by CCK8 assays after 24 h and 48 h of 200 μM nootkatone treatment (n = 5/group, one-way ANOVA, Tukey’s test was performed for the multiple comparison). (**D**) Typical images of U251 cells transfected with sh-ATF4 constructs and then treated with 200 μM nootkatone for 24 h in transwell migration assay. Scale bar, 200 µm. (**E**) The numbers of migrated cells were counted in representative high-power fields per transwell plate as shown in (**D**) (n = 5/group, one-way ANOVA, Tukey’s test was performed for the multiple comparison). Data were mean ± SEM. *^*^P < 0.05, ^**^P < 0.01.*
